# Knowledge of Herpes Zoster Virus and Its Vaccines Among Older Adults in Jazan Province, Saudi Arabia: A Cross-Sectional Study

**DOI:** 10.7759/cureus.68726

**Published:** 2024-09-05

**Authors:** Abdulaziz H Alhazmi, Hassan Jaafari, Anwar H Hufaysi, Alwaleed K. Alhazmi, Fahad Harthi, Taif Khalid Mohammed Hakami, Reem T Hadadi, Naif Gharwi, Raghad H. Bajawi, Ehab Farouq Hakami, Nouf M. Hakami, Mohammed M Elfaki

**Affiliations:** 1 Microbiology, Jazan University, Jazan, SAU; 2 College of Medicine, Jazan University, Jazan, SAU; 3 Faculty of Medicine, Jazan University, Jazan, SAU

**Keywords:** herpes zoster, immunization, jazan, knowledge, shingles, vaccine

## Abstract

Introduction

Herpes zoster (HZ) is caused by the reactivation of the varicella-zoster virus (VZV) and can lead to complications such as postherpetic neuralgia. Although vaccines are available to prevent HZ, the level of concern about HZ and its vaccines in our region remains unknown. This study assessed the knowledge, attitudes, and practices regarding HZ and the HZ vaccines among adults aged ≥50 years in Jazan, Saudi Arabia.

Methods

A cross-sectional study was conducted using a questionnaire distributed to adults aged ≥50 years and data were collected on demographics, knowledge of HZ and its vaccines, attitudes, and practices. Multiple logistic regression examined factors associated with knowledge levels.

Results

Of 295 participants, 58% and 67.5% had low knowledge of HZ and its vaccines, respectively. Knowledge of HZ significantly differed by age, education, and occupation. Only 50% knew HZ affects nerves and skin and 28.8% knew HZ can be transmitted between individuals. Knowledge of the vaccine significantly differed by gender and information source.

Conclusions

This population has substantial knowledge gaps regarding HZ and vaccination. Targeted educational initiatives are needed to promote greater awareness of HZ immunization, especially for higher-risk groups like older adults and females.

## Introduction

Herpes zoster (HZ), commonly known as shingles, is a disease caused by the reactivation of the varicella-zoster virus (VZV), the same virus that causes chickenpox [1]. VZV is an enveloped double-stranded DNA virus belonging to the Herpesviridae family [1]. It causes two distinct diseases at different stages of life. The initial exposure to VZV typically results in chickenpox, predominantly seen in children or adults who were not infected during childhood [1,2]. After the initial infection, the virus remains dormant in the dorsal sensory root ganglia and cranial nerves. Reactivation of the virus, often triggered by immunosuppression, leads to HZ [3], a condition that can severely affect the neurological system and result in complications such as postherpetic neuralgia (PHN), encephalitis, meningitis, and ocular involvement [4,5]. The refractory nature of PHN to treatment significantly impacts quality of life [6-8].

The prevalence of HZ has been increasing over the past few decades, particularly among older adults, with an annual incidence rate of 3-5/1000 in the general population and 5-10/1000 among those aged over 50 years [9,10]. More than 95% of individuals over 50 years old are at risk of developing HZ due to immunosenescence, making them more susceptible to PHN following an HZ infection [11,12]. Vaccination against HZ has been shown to bolster the immune response, with two licensed vaccines available to prevent the disease: the live attenuated zoster vaccine (ZVL) and the recombinant zoster vaccine (RZV). ZVL, also known as the zoster vaccine live, is a live attenuated vaccine that uses a weakened form of the varicella-zoster virus to stimulate the immune system to mount a response against the virus, thereby boosting immunity and reducing the risk of reactivation as shingles. However, its efficacy tends to decrease with age, particularly in individuals over 70 years old [13,14]. In comparison, RZV, FDA-approved in 2017, is a recombinant, adjuvanted vaccine that utilizes a glycoprotein E antigen and an adjuvant system (AS01B) to enhance the immune response. RZV shows superior efficacy (56.9% for the first dose, and 70.1% for the second) and is recommended for adults aged ≥50 years [15,16], with reported efficacy against HZ and PHN at 91.3% and 88.8%, respectively [17,18].

Understanding public knowledge, attitudes, and practices regarding HZ and its vaccine is crucial for addressing these barriers. Despite the significance of this issue, there is limited data on public awareness and knowledge of HZ and VZV, particularly in our region. Studies conducted in other countries have highlighted gaps in knowledge and awareness, which may influence vaccine uptake [19-21]. Therefore, this study aims to assess the knowledge, attitudes, and practices related to HZ and its vaccines among adults aged 50 years and older in the Jazan region of Saudi Arabia.

## Materials and methods

Study design, setting, and participants

This cross-sectional observational study was conducted in the Jazan region of Saudi Arabia, with data collected from March 2, 2023, to August 1, 2023. The Jazan region, located in the southwest of Saudi Arabia, has a population of around 1.6 million people. The study population included adults aged 50 years and older who were permanent residents of the Jazan region during the study period. Participants were included if they were aged 50 years or older, able to provide informed consent, and willing to participate in the study by completing the survey. We excluded individuals who refused to participate, those who resided outside the Jazan region during the study period, and those with a known diagnosis of cognitive impairment or other conditions that would preclude them from providing reliable self-reported data. Additionally, bedridden or severely ill individuals, for whom participation in the survey was impractical or impossible, were also excluded.

Sample size and sampling method

The sample size for this study was determined using the Raosoft sample size calculator (Raosoft, Inc., Seattle, WA), and it was found that 271 participants were required to achieve a 90% confidence interval, a 5% margin of error, and 50% prevalence as no prior study was conducted in this region. A simple random sampling technique was used to select participants from the Jazan population.

Data collection and study tool

The data were collected through a self-administered questionnaire (Appendix), which was developed following a comprehensive review of related articles and consultation with an expert in the field [18-20]. The questionnaire was crafted in Arabic and distributed online to the targeted population via a link shared by trained data collectors. These data collectors played a crucial role in identifying eligible participants, particularly in community settings like clinics and medical centers, and then guiding them on how to access and complete the questionnaire online. The distribution strategy was non-random; instead, it involved targeted outreach to ensure that the questionnaire reached a representative sample of the older adult population in Jazan.

The questionnaire was structured into three sections: socio-demographic characteristics of participants, general questions regarding HZ knowledge, and specific questions related to HZ. The knowledge level about HZ and its vaccines was assessed using a total of eight questions. Participants were classified as having low knowledge if they correctly answered four or fewer questions, and as having high knowledge if they answered five or more questions correctly. The cut-off of four correct answers was chosen based on preliminary analyses and expert consultation, suggesting that fewer than five correct answers indicated a substantial gap in understanding the key concepts related to HZ and its vaccines.

Before the main study, a pilot study was conducted with 20 participants to test the Arabic translation of the data collection tool. Following the pilot, several enhancements and reorganization of questions were implemented to improve the clarity and usability of the questionnaire. The final version of the questionnaire demonstrated good internal consistency, with a Cronbach’s alpha of 0.87, and test-retest reliability, with a correlation efficiency of 0.80.

Ethical consideration

Our study received approval from the Scientific Research Ethics Committee (REC) at Jazan University in Saudi Arabia (reference number: REC-44/07/515; date: January 30, 2023). Informed consent was obtained from all participants, and their information was maintained confidentially, with the data solely utilized for scientific purposes.

## Results

Through an online questionnaire and interviews, 295 responses were gathered out of 301 potential participants, with six individuals declining to participate or refusing to provide consent. The findings revealed that the participants' responses varied based on factors such as age and educational background. However, there were no significant differences observed based on gender or occupation. Specifically, 46.1% (n = 136) of the participants belonged to the 50-54 years age group, while 24.1% (n = 71) were in the 55-59 years age group. The 60-64 years age group accounted for 14.6% (n = 43), and individuals aged over 65 years made up 15.3% (n = 45). In terms of gender distribution, 51.9% (n = 153) of the participants were female, while 48.1% (n = 142) were male. As for educational backgrounds, the majority of participants had completed a college but not a bachelor’s degree, representing 40.7% (n = 120). A small proportion, approximately 1.4% (n = 4) held a bachelor's degree. Of the participants, 22% (n = 65) had finished secondary school. In terms of employment, 28.8% (n = 85) were employed in non-health sectors whereas 4.7% (n = 14) were in the healthcare sector. Of the participants, 6.8% (n = 20) were self-employed, and 29.5% (n = 87) had retired (Table 1).

**Table 1:** Demographic distribution of the participants (n = 295).

Variables		N	%
Age groups	50-54	136	46.1%
	55-59	71	24.1%
	60-64	43	14.6%
	>65	45	15.3%
Gender	Male	142	48.1%
	Female	153	51.9%
Education level	Less than high school	106	35.9%
	High school	65	22.0%
	Diploma	120	40.7%
	Bachelor's degree	4	1.4%
Occupation	Healthcare sector employee	14	4.7%
	Non-healthcare sector employee	85	28.8%
	Student	3	1.0%
	Self-employed	20	6.8%
	Retired	87	29.5%
	Non-employed	86	29.2%

Table 2 presents the responses to questions regarding the knowledge of HZ. For the answers to the question about what is HZ, 56.6% (n = 167) of the participants correctly knew it as a disease that mostly affects nerves and skin, while 1.4% (n = 4) recognized it as a disease that affects the brain, 0.7% (n = 2) as a disease that affects the lung, and 41.4% (n = 122) were not sure about the answer. About 26.8% (n = 79) of the participants think that HZ is common in Saudi Arabia, 22.0% (n = 65) did not, and 51.2% (n = 151) were not sure. The majority (65.1%, n = 191) were not sure if it could be infected more than once. Only 21% (n = 62) realize it. Regarding age, 38% (n = 113) recognize the age of 50 and above as the most susceptible to the disease. Only 19% were aware that HZ cannot be transmitted from one person to another. The skin rash was the most identified symptom (53%, n = 158), followed by neuralgia (39%, n = 115). However, other symptoms such as vision and hearing problems (86.1%, n = 254) and headache (80.7%, n = 238) were not recognized by most of them.

**Table 2:** Responses to questions regarding the knowledge of HZ and its vaccines. * Right answer.

Questions		N	%
Based on what you've heard or know, what is herpes zoster (shingles)?	A disease that mostly affects nerves and skin*	167	56.6%
	A disease that mostly affects the brain	4	1.40%
	A disease that affects mostly the lungs	2	0.70%
	Not sure	122	41.4%
Do you think herpes zoster (shingles) is common in Saudi Arabia?	Yes	79	26.8%
	No	65	22.0%
	Not sure	151	51.2%
Can a person get herpes zoster (shingles) more than once?	Yes*	62	21.0%
	No	41	13.9%
	Not sure	192	65.1%
Which age group do you think is most susceptible to getting herpes zoster (shingles)?	Under 40 years old	26	8.80%
	41-49 years old	24	8.10%
	50 years old and above*	113	38.3%
	Not sure	132	44.7%
Do you know any symptoms of herpes zoster (shingles)?	Yes	129	43.7%
	No	92	31.2%
	Not sure	74	25.1%
Neuralgia	Yes*	115	39.0%
Skin rash	Yes*	158	53.6%
Vision or hearing problems	Yes*	41	13.9%
Headache	Yes*	57	19.3%
Do you think herpes zoster (shingles) can be cured?	No*	14	4.70%
Do you believe that herpes zoster (shingles) can be prevented?	Yes*	154	52.0%
Responses to questions regarding the knowledge of the HZ vaccine			
Do you believe that the herpes zoster (shingles) vaccine is safe and effective?	Yes*	90	30.0%
Do you think that the herpes zoster (shingles) vaccine has side effects?	Yes*	121	40.0%
Headache	Yes*	95	32.2%
Pain at the injection site	Yes*	110	37.3%
Swelling at the injection site	Yes*	96	32.5%
Redness at the injection site	Yes*	92	31.2%
Fatigue	Yes*	102	34.6%
Fever	Yes*	94	31.9%
Do you believe that receiving the herpes zoster (shingles) vaccine can be beneficial even if someone has had shingles before?	Yes*	88	29.8%

Study respondents showed various age groups with high knowledge (52.2%) at the age of 50-54 years, followed by 39.4% and 35.6% of age groups 55-59 and >65 years, and only 20.9% of age groups 60-64 years. There was no significant difference in knowledge across genders (p = 0.757). The results showed a significant difference in HZ knowledge across education levels (p = 0.001). Most participants with a college degree less than a bachelor had high knowledge (55.8%); moreover, the participants with a bachelor's degree had equal knowledge, while those with a high school diploma or equivalent or less than high school seemed to have low knowledge (61.5% and 70.8%). A significant difference in knowledge was found across occupations (p = 0.012). Most healthcare sector employees had high knowledge (78.6%), followed by students (66.7%), and almost half of non-healthcare sector employees and retirees had high knowledge (47.1% and 43.7%). Only 31.4% and 30.0% of homemakers and self-employed people had high knowledge. Employment in healthcare may increase HZ awareness.

The research findings reveal notable disparities in the sources of knowledge acquisition between respondents, characterized by high and low levels of familiarity with the HZ. Among those with high knowledge, a substantial proportion, specifically 68.4%, demonstrated a preference for the Ministry of Health as their primary source of information, while 60.3% turned to internet sites and social networking platforms for their initial knowledge about HZ. Additionally, 57.7% of high-knowledge respondents indicated a strong reliance on their families and friends for insights into HZ, with 52.3% citing television as a significant information source. Notably, 33.3% of high-knowledge respondents obtained their understanding of HZ through their prior experiences with the infection, and radio played a lesser role, with only 20.0% of respondents utilizing it as an information source. Low-knowledge respondents exhibited a markedly different pattern of knowledge sources. A substantial 80.0% of low-knowledge respondents identified the radio as their primary source of information regarding HZ, followed closely by 66.7% who relied on their previous encounters with the virus. Television served as a significant source for 47.7% of low-knowledge respondents, while 42.3% attributed their knowledge of HZ to their families and friends. In contrast to high-knowledge respondents, the Ministry of Health and internet sites and social networking platforms were the least preferred sources, with only 31.6% and 39.7%, respectively, choosing these channels for obtaining information about the HZ virus (Table 3).

**Table 3:** Knowledge level of herpes zoster (HZ) across demographic groups. * The alpha criterion for the p-value was set to 0.05.

Variables		Knowledge of HZ				P-value
		Low knowledge (n = 171, 57.97%)		High knowledge (n = 124, 42.03%)		
		N	%	N	%	
Age groups	50-54	65	47.8%	71	52.2%	0.002*
	55-59	43	60.6%	28	39.4%	
	60-64	34	79.1%	9	20.9%	
	>65	29	64.4%	16	35.6%	
Gender	Male	81	57.0%	61	43.0%	0.757
	Female	90	58.8%	63	41.2%	
Education level	Less than high school	75	70.8%	31	29.2%	0.001*
	High school	41	63.1%	24	36.9%	
	Diploma	53	44.2%	67	55.8%	
	Bachelor's degree	2	50.0%	2	50.0%	
Occupation	Healthcare sector employee	3	21.4%	11	78.6%	0.012*
	Non-healthcare sector employee	45	52.9%	40	47.1%	
	Student	1	33.3%	2	66.7%	
	Self-employed	14	70.0%	6	30.0%	
	Retired	49	56.3%	38	43.7%	
	Non-employed	59	68.6%	27	31.4%	
Source of Knowledge						
Family/friends	No	118	69.0%	53	31.0%	<0.0001*
	Yes	52	42.3%	71	57.7%	
TV	No	149	59.6%	101	40.4%	0.141
	Yes	21	47.7%	23	52.3%	
Internet sites and social networking	No	143	63.3%	83	36.7%	0.001*
	Yes	27	39.7%	41	60.3%	
The radio	No	165	57.3%	123	42.7%	0.308
	Yes	4	80.0%	1	20.0%	
Ministry of Health	No	152	64.1%	85	35.9%	<0.0001*
	Yes	18	31.6%	39	68.4%	
Previous infection	No	167	57.8%	122	42.2%	0.663
	Yes	4	66.7%	2	33.3%	

The results showed no significant difference in HZ vaccine knowledge across age groups 50-54, 55-59, 60-64, and >65 years (p = 0.770). The percentage with high knowledge ranged from 31.0% to 39.5% across age groups. Males had significantly higher knowledge than females (39.4% vs. 26.1, p = 0.015). There was no significant difference in knowledge across education levels (p = 0.317). No significant difference in knowledge was found across occupations (p = 0.118). However, healthcare workers had higher knowledge (64.3% high) versus other groups. Employment in healthcare may be associated with increased HZ vaccine knowledge. The study found statistically significant associations (p < 0.05) between exposure to television, the internet, and the Ministry of Health and higher levels of knowledge about the HZ vaccine. Increased circulation by media sources, particularly official outlets such as the Ministry of Health, appears to correlate with greater knowledge of the HZ vaccine. These results suggest that media exposure, especially from authoritative sources, may play an important role in disseminating knowledge and raising public awareness related to vaccination options such as the HZ vaccine (Table 4).

**Table 4:** Knowledge of herpes zoster (HZ) vaccine across demographic groups. * The alpha criterion for the p-value was set to 0.05.

Variables		Knowledge of HZ vaccination				P-value
		Low knowledge (n = 199, 67.46%)		High knowledge (n = 96, 32.54%)		
		n	%	n	%	
Age groups	50-54	93	68.4%	43	31.6%	0.770
	55-59	49	69.0%	22	31.0%	
	60-64	26	60.5%	17	39.5%	
	>65	31	68.9%	14	31.1%	
Gender	Male	86	60.6%	56	39.4%	0.015*
	Female	113	73.9%	40	26.1%	
Education level	Less than high school	75	70.8%	31	29.2%	0.317
	High school	40	61.5%	25	38.5%	
	Diploma	80	66.7%	40	33.3%	
	Bachelor's degree	4	100.0%	0	0.0%	
Occupation	Healthcare sector employee	5	35.7%	9	64.3%	0.118
	Non-healthcare sector employee	59	69.4%	26	30.6%	
	Student	1	33.3%	2	66.7%	
	Self-employed	13	65.0%	7	35.0%	
	Retired	60	69.0%	27	31.0%	
	Non-employed	61	70.9%	25	29.1%	
Family/friends	No	113	66.1%	58	33.9%	0.585
	Yes	85	69.1%	38	30.9%	
TV	No	177	70.8%	73	29.2%	0.003*
	Yes	21	47.7%	23	52.3%	
Internet sites and social networking	No	159	70.4%	67	29.6%	0.045*
	Yes	39	57.4%	29	42.6%	
The radio	No	193	67.0%	95	33.0%	0.118
	Yes	5	100.0%	0	0.0%	
Ministry of Health	No	171	72.2%	66	27.8%	<0.0001*
	Yes	27	47.4%	30	52.6%	
Previous infection	No	196	67.8%	93	32.2%	0.356
	Yes	3	50.0%	3	50.0%	

The regression analysis (Table 5) reveals that demographic characteristics (age, gender, education level, or occupation) do not significantly affect the level of HZ knowledge. Conversely, the source of information plays a pivotal role. Participants who did not obtain their information from family, friends, internet sites, or social networks were more likely to have lower HZ knowledge (p < 0.001, p = 0.010). Those who did not listen to the radio had a significantly higher level of HZ knowledge (p = 0.041). Participants not learning from the Ministry of Health also exhibited lower knowledge levels (p = 0.001).

**Table 5:** Factors associated with lower knowledge of herpes zoster (HZ) among the participants of the study using multiple logistic regression. a. The reference category: low knowledge. * The alpha criterion for the p-value was set to 0.05.

Variables		P-value	Odds ratio	95% confidence interval for odds ratio	
				Lower bound	Upper bound
	Intercept	0.934			
Age group	50-54	0.386	1.511	0.594	3.841
	55-59	0.834	1.106	0.430	2.844
	60-64	0.283	0.549	0.183	1.643
	>65	.	.	.	.
Gender	Male	0.787	1.110	0.523	2.356
	Female	.	.	.	.
Education level	Less than high school	0.965	0.947	0.083	10.847
	High school diploma or equivalent	0.955	0.933	0.083	10.455
	Some college or associate's degree	0.738	1.505	0.137	16.486
	Bachelor's degree	.	.	.	.
Occupation	Healthcare sector employee	0.244	2.883	0.485	17.130
	Non-healthcare sector employee	0.769	0.852	0.291	2.491
	Student	0.348	4.337	0.202	92.909
	Self-employed	0.919	1.079	0.249	4.683
	Retired	0.245	1.815	0.665	4.956
	Non-employed	.	.	.	.
Source of knowledge	Family/friends = No	0.000*	0.205	0.112	0.377
	Family/friends = Yes	.	.	.	.
	TV = No	0.445	0.723	0.314	1.662
	TV = Yes	.	.	.	.
	Internet sites and social networking = No	0.010*	0.390	0.190	0.802
	Internet sites and social networking = Yes	.	.	.	.
	The radio = No	0.041	16.182	1.120	233.719
	The radio = Yes	.	.	.	.
	Ministry of Health = No	0.001*	0.257	0.116	0.567
	Ministry of Health = Yes	.	.	.	.
Previous infection	Previous infection = No	0.379	0.440	0.071	2.745
	Previous infection = Yes	.	.	.	.

Family and friends are considered the major sources of information about HZ by 41.7% (n = 123) of participants, followed by the internet by 23.1% (n = 68), Ministry of Health by 19.3% (n = 57), TV by 14.9% (n = 44), previous infection by 2% (n = 6), and radio by 1.7% (n = 5). The percentage of participants' attitudes and practices and their concern about getting shingles or HZ was 62.7%, and only 17.6% were recommended to get the HZ vaccine by a healthcare provider. In addition, 48.8% of the participants were willing to get the HZ vaccine if recommended, and 81.7% of them, if recommended to get the vaccine, would ask their healthcare provider or another healthcare professional for more information about the HZ vaccine. However, only 29.9% of participants believe that the HZ (shingles) vaccine is safe and effective.

## Discussion

This study aimed to assess the knowledge and attitudes surrounding HZ and the HZ vaccine among a sample of adults of 50 years or older in the southwestern region of Saudi Arabia, where limited data exist about this topic. The importance of this study stemmed from the availability of the HZ shingles vaccine for the population of 50 years or older in Saudi Arabia, as announced recently (2022) by the Ministry of Health in Saudi Arabia [21]. Yet, notable findings carry implications for public health initiatives in Saudi Arabia and mostly align with national and international observations [22-28]. The results revealed that 58% of respondents exhibited a low level of knowledge regarding HZ (Table 3), and even 67% demonstrated limited knowledge of the HZ vaccine. Aligned with other observations [25-28], the study identified significant variations in HZ knowledge based on age, educational background, occupation, and source of information (Table 3). Univariate analysis showed individuals aged 50-54 years and those with a college education and being healthcare workers displayed higher levels of knowledge compared to older age groups and individuals with lower educational backgrounds, aligned with another study from the central region of the country [27]. Further, accessing information through family, friends, or Ministry of Health websites was strongly correlated with higher knowledge, with internet sites and radio also emerging as significant information sources [25]. Thus, we believe that the Ministry of Health is recommended to enhance its online and offline presence as it was done during the COVID-19 pandemic [29], develop user-friendly platforms, and collaborate with radio channels and other television networks to disseminate accurate and accessible health information about shingles and its serious outcomes. This proactive approach will not only empower individuals seeking information but also contribute to a more informed and health-conscious community.

While 44% of the respondents in this study demonstrated adequate knowledge of common HZ symptoms such as rash and neuralgia, the recognition of less prevalent symptoms like vision or hearing problems was notably lower, ranging from 13% to 19%. This finding aligns with other national and international findings [22-28], exemplified by Modin et al. (2019), who observed limited symptom recognition among the general public in their study population [23]. Only a minority of participants in their study could identify vision or hearing problems as potential HZ symptoms, mirroring the current study where vision problems were recognized by a small fraction of respondents (13.9%) [23]. The consistent lack of knowledge regarding the full spectrum of HZ manifestations across both studies suggests a general limitation in recognition across diverse populations. As noted by Modin et al. (2019), this restricted symptom recognition could potentially lead to delayed medical care if individuals do not associate rare but serious HZ symptoms with the condition [23]. Consistent with their recommendations and others [22-28], the findings of the present study emphasize the need for educational materials on HZ to emphasize the full range of possible manifestations beyond the classic rash and neuralgia. Increasing awareness of less common but significant symptoms could facilitate prompt healthcare-seeking behavior among affected individuals. The advantages derived from the early identification of shingles symptoms extend beyond facilitating prompt and targeted medical intervention. They encompass the mitigation of symptom severity and duration, prevention of complications, and the reduction of the risk of disease transmission to others. Notably, this recognition could be broadened to encompass the enhancement of public awareness, given that a significant proportion of participants relied on information from friends and family to comprehend the varied manifestations and potential complications associated with shingles. Acknowledging the reliance on informal sources for information highlights the need for comprehensive educational campaigns to reach a wider audience and improve knowledge dissemination about the diverse symptoms of shingles. Addressing this knowledge gap is crucial to fostering a proactive and informed approach to healthcare-seeking behavior and ensuring a more robust public understanding of shingles and its implications.

Knowledge pertaining to the HZ vaccine was found to be lower than that of HZ itself and, as reported by others, exhibited significant variations based on gender and information sources [25-28]. Yet, about 50% will get the vaccine once recommended and 80% will get it after getting the right information from a healthcare professional. It seems that the Ministry of Health websites significantly contributed to attitudes and practices enhancement, notably after the availability of the vaccine [21]. Our findings indicated that knowledge of vaccines was higher among younger age groups, individuals with higher educational attainment, healthcare workers, and those who accessed information from family, friends, or the Ministry of Health. This observation aligns with expectations, as older individuals and those with lower educational levels typically have limited access to health information and online resources in general. It indicates the pivotal roles healthcare providers and official health sources can play in disseminating accurate information [21,22]. Consistent with previous research, knowledge concerning the HZ vaccine was found to be even lower, with only 32.5% exhibiting high knowledge. In comparison with international findings, a European study reported vaccine awareness at a mere 11.8% [23]. Our findings revealed that women, older adults, and those not exposed to information through television or online sources possessed significantly lower vaccine knowledge, providing valuable insights into key demographic groups that should be targeted in educational interventions.

This study faces several limitations. Primarily, the way of survey distribution introduces the potential for selection bias, as it inherently favors respondents with internet access, potentially excluding perspectives from less digitally engaged individuals. This approach, coupled with the dependence on self-reported data, introduces risks of recall and social desirability biases, wherein participants may unintentionally or intentionally misrepresent their experiences or viewpoints. The cross-sectional nature of the study further confines its scope, capturing only a momentary snapshot and preventing an analysis of causal relationships or temporal changes in attitudes and awareness. Moreover, the assumption of a homogenous target population may overlook nuanced differences in knowledge and attitudes influenced by unaccounted factors such as socio-economic or cultural diversity. Finally, while the response rate is considerable, it falls short of the optimal sample size with a low CI of 90%, potentially compromising the study's statistical robustness and the generalizability of its conclusions. Overall, our results are consistent with previous research demonstrating substantial gaps in HZ and vaccine awareness globally. Public health initiatives should seek to address these knowledge deficits, particularly among vulnerable demographic groups. Expanding health education through multiple channels, including providers, traditional media, and online platforms, may help boost knowledge levels in this population. Further research is still needed to identify the most effective strategies for improving awareness.

## Conclusions

In conclusion, we assessed the level of awareness and knowledge regarding HZ and its vaccines among older people in the Jazan region. Our findings revealed that a majority of participants demonstrated awareness of HZ, while awareness of the vaccine varied across different age groups. Many participants expressed concerns about getting HZ, and a significant portion exhibited a willingness to receive the vaccine if recommended. Based on these results, healthcare providers must make a greater effort to raise awareness about HZ and promote the importance of recommending the vaccine among individuals over 50 years of age. This proactive approach can significantly reduce the disease burden and potential complications associated with HZ.

## Appendices

Questionnaire used in the study

Title: Assessment of Knowledge Regarding Herpes Zoster (Shingles) and the Willingness to Take Its Vaccine Among Residents of the Jazan Region

Consent question:

• Do you agree to participate in this study? - Yes - No

Demographics:

• Age: - (Specify age)

• Gender: - Male - Female

• Educational level: - Below high school - High school - Diploma or Bachelor's degree - Postgraduate

• Occupation: - Healthcare sector employee - Non-healthcare sector employee - Student - Self-employed - Retired - Homemaker

General knowledge about herpes zoster (shingles):

• Have you heard about herpes zoster (shingles)? - Yes - No

• If yes, how did you hear about it? - Family - Friends - Television - Internet and social networking - Radio - Ministry of Health - Other (please specify)

Specific questions regarding herpes zoster:

• Based on what you’ve heard or know, what is herpes zoster (shingles)? - A disease that affects the nerves and skin - A disease that affects the brain - A disease that affects the lungs - I don't know

• Do you think herpes zoster (shingles) is common in Saudi Arabia? - Yes - No - I don't know

• Have you ever been infected with herpes zoster (shingles) in the past? - Yes - No - I don't know

• Do you know any of the symptoms of herpes zoster (shingles)? - Yes - No - I don't know

• Do you think you can get infected with herpes zoster (shingles) more than once? - Yes - No - I don't know

• Have you ever known someone who was infected with herpes zoster (shingles)? - Yes - No - I don't know

• Based on what you’ve heard or know, do you think herpes zoster (shingles) can be transmitted from person to person? - Yes - No - I don't know

• Which age group do you think is most at risk of getting herpes zoster (shingles)? - Under 40 years - Between 41 and 49 years - 50 years and older - I don't know

• Are you concerned about getting herpes zoster (shingles)? - Yes - No - I don't know

• What do you think is the most common complication of herpes zoster (shingles)? - Nerve pain - Skin rash - Eye problems or vision impairment - Hearing loss - Depression - Headache - I don't know

• Do you think herpes zoster (shingles) can be treated? - Yes - No - I don't know

• Do you think herpes zoster (shingles) can be prevented? - Yes - No - I don't know

General knowledge about the herpes zoster vaccine:

• Have you heard about the herpes zoster (shingles) vaccine? - Yes - No

• If yes, how did you hear about the vaccine? - Healthcare practitioner - Family - Friends - Advertisements - Television - Internet - Other (please specify)

• Has your doctor or any other healthcare practitioner ever recommended that you take the herpes zoster (shingles) vaccine? - Yes - No

• Are you willing to take the herpes zoster (shingles) vaccine if asked to do so? - Yes - No - Not sure

• If asked to take the vaccine, would you ask your doctor or any other healthcare practitioner for more information about the herpes zoster (shingles) vaccine? - Yes - No - I don't know

• Do you know anyone among your acquaintances who have received the herpes zoster (shingles) vaccine? - Yes - No - I don't know

• Do you think the herpes zoster (shingles) vaccine is safe and effective? - Yes - No - I don't know

• Do you think the herpes zoster (shingles) vaccine has side effects? - Yes - No - I don't know

• If yes, what do you think is the most common side effect of the vaccine? - Headache - Pain at the injection site - Swelling at the injection site - Redness at the injection site - Fatigue - Fever - I don't know - Other (please specify)

• Do you think the herpes zoster (shingles) vaccine can be taken even if the person has had herpes zoster before? - Yes - No - Not sure

## References

[1] Straus SE, Ostrove JM, Inchauspé G, Felser JM, Freifeld A, Croen KD, Sawyer MH (1988). Varicella-zoster virus infections: biology, natural history, treatment, and prevention. Ann Intern Med.

[2] Cohen JI (2013). Herpes zoster. N Engl J Med.

[3] Blair RJ (2019). Varicella Zoster virus. Pediatr Rev.

[4] Koshy E, Mengting L, Kumar H, Jianbo W (2018). Epidemiology, treatment and prevention of herpes zoster: a comprehensive review. Indian J Dermatol Venereol Leprol.

[5] Stein AN, Britt H, Harrison C, Conway EL, Cunningham A, Macintyre CR (2009). Herpes zoster burden of illness and health care resource utilisation in the Australian population aged 50 years and older. Vaccine.

[6] Drolet M, Brisson M, Schmader KE (2010). The impact of herpes zoster and postherpetic neuralgia on health-related quality of life: a prospective study. CMAJ.

[7] Tseng HF, Smith N, Harpaz R, Bialek SR, Sy LS, Jacobsen SJ (2011). Herpes zoster vaccine in older adults and the risk of subsequent herpes zoster disease. JAMA.

[8] John AR, Canaday DH (2017). Herpes zoster in the older adult. Infect Dis Clin North Am.

[9] Kawai K, Gebremeskel BG, Acosta CJ (2014). Systematic review of incidence and complications of herpes zoster: towards a global perspective. BMJ Open.

[10] van Oorschot D, Vroling H, Bunge E, Diaz-Decaro J, Curran D, Yawn B (2021). A systematic literature review of herpes zoster incidence worldwide. Hum Vaccin Immunother.

[11] Johnson RW, Alvarez-Pasquin MJ, Bijl M (2015). Herpes zoster epidemiology, management, and disease and economic burden in Europe: a multidisciplinary perspective. Ther Adv Vaccines.

[12] Harbecke R, Cohen JI, Oxman MN (2021). Herpes zoster vaccines. J Infect Dis.

[13] Dooling KL, Guo A, Patel M, Lee GM, Moore K, Belongia EA, Harpaz R (2018). Recommendations of the advisory committee on immunization practices for use of herpes zoster vaccines. MMWR Morb Mortal Wkly Rep.

[14] Izurieta HS, Wu X, Forshee R (2021). Recombinant zoster vaccine (Shingrix): real-world effectiveness in the first 2 years post-licensure. Clin Infect Dis.

[15] Yancey KB (2016). Commentary regarding: Efficacy of an adjuvanted herpes zoster subunit vaccine in older adults. H Lal, AL Cunningham, O Godeaux et al., N Engl J Med 372:2087-2096, 2015. Dermatol Ther.

[16] Cunningham AL, Lal H, Kovac M (2016). Efficacy of the herpes zoster subunit vaccine in adults 70 years of age or older. N Engl J Med.

[17] López-Fauqued M, Co-van der Mee M, Bastidas A (2021). Safety profile of the adjuvanted recombinant zoster vaccine in immunocompromised populations: an overview of six trials. Drug Saf.

[18] Elkin Z, Cohen EJ, Goldberg JD (2013). Studying physician knowledge, attitudes, and practices regarding the herpes zoster vaccine to address perceived barriers to vaccination. Cornea.

[19] Ecarnot F, Pedone C, Cesari M, Maggi S, Antonelli Incalzi R (2020). Knowledge about vaccines and vaccination in older people: results of a national survey by the Italian Society for Gerontology & Geriatrics. Vaccine.

[20] Marx GE, Chase J, Jasperse J (2017). Public health economic burden associated with two single measles case investigations — Colorado, 2016-2017. MMWR Morb Mortal Wkly Rep.

[21] (2022). Shingles vaccine available in primary care centers, says Health Ministry. https://saudigazette.com.sa/article/625548.

[22] Jacobs W, Amuta AO, Jeon KC (2017). Health information seeking in the digital age: an analysis of health information seeking behavior among US adults. Cogent Soc Sci.

[23] Modin D, Jørgensen ME, Gislason G (2019). Influenza vaccine in heart failure: cumulative number of vaccinations, frequency, timing, and survival: a Danish nationwide cohort study. Circulation.

[24] Di Giuseppe G, Pelullo CP, Napoli A, Napolitano F (2023). Willingness to receive Herpes Zoster vaccination among adults and older people: a cross sectional study in Italy. Vaccine.

[25] AlMuammar S, Albogmi A, Alzahrani M, Alsharef F, Aljohani R, Aljilani T (2023). Herpes zoster vaccine awareness and acceptance among adults in Saudi Arabia: a survey-based cross-sectional study. Trop Dis Travel Med Vaccines.

[26] Alhothali OS, Alhothali AS, Hanif AA, Bondagji MF, Aljabri HM, Goweda R (2023). A cross-sectional study of the knowledge, practice, and attitude towards herpes zoster vaccination among the general population in the western region of Saudi Arabia. Cureus.

[27] Al-Orini D, Alshoshan AA, Almutiri AO (2023). Acceptability of herpes zoster vaccination among patients with diabetes: a cross-sectional study in Saudi Arabia. Vaccines (Basel).

[28] Alleft LA, Alhosaini LS, Almutlaq HM, Alshayea YM, Alshammari SH, Aldosari MA, Alateeq FA (2023). Public knowledge, attitude, and practice toward Herpes zoster vaccination in Saudi Arabia. Cureus.

[29] Assiri A, Al-Tawfiq JA, Alkhalifa M (2021). Launching COVID-19 vaccination in Saudi Arabia: lessons learned, and the way forward. Travel Med Infect Dis.

